# Identification of prognostic values of the transcription factor-CpG-gene triplets in lung adenocarcinoma: A narrative review

**DOI:** 10.1097/MD.0000000000032045

**Published:** 2022-12-16

**Authors:** Duohuang Lian, Luoyu Lian, Dehua Zeng, Meiqing Zhang, Mengmeng Chen, Yaming Liu, Wenmin Ying, Shunkai Zhou

**Affiliations:** a Department of Thoracic and Cardiac Surgery, The 900th Hospital of The Joint Logistics Support Force of The People's Liberation Army, Fuzhou City, Fujian Province, China; b Department of Thoracic Surgery, Quanzhou First Hospital Affiliated to Fujian Medical University, Quanzhou City, Fujian Province, China; c Department of Pathology, The 900th Hospital of The Joint Logistics Support Force of The Chinese People's Liberation Army, Fuzhou City, Fujian Province, China; d Department of Radiotherapy, Fuding Hospital, Fuding City, Fujian Province, China.

**Keywords:** DNA methylation, lung adenocarcinoma, prognosis, transcription factor

## Abstract

Abnormal DNA methylation can regulate carcinogenesis in lung adenocarcinoma (LUAD), while transcription factors (TFs) mediate methylation in a site-specific manner to affect downstream transcriptional regulation and tumor progression. Therefore, this study aimed to explore the TF-methylation-gene regulatory relationships that influence LUAD prognosis.

**Methods:**

Differential analyses of methylation sites and genes were generated by integrating transcriptome and methylome profiles from public databases. Through target gene identification, motif enrichment in the promoter region, and TF prediction, TF-methylation and methylation-gene relation pairs were obtained. Then, the prognostic TF-methylation-gene network was constructed using univariate Cox regression analysis. Prognostic models were constructed based on the key regulatory axes. Finally, Kaplan-Meier curves were created to evaluate the model efficacy and the relationship between candidate genes and prognosis.

**Results:**

A total of 1878 differential expressed genes and 1233 differential methylation sites were screened between LUAD and normal samples. Then 10 TFs were predicted to bind 144 enriched motifs. After integrating TF-methylation and methylation-gene relations, a prognostic TF-methylation-gene network containing 4 TFs, 111 methylation sites, and 177 genes was constructed. In this network, *ERG*-cg27071152-*MTURN* and *FOXM1*-cg19212949-*PTPR* regulatory axes were selected to construct the prognostic models, which showed robust abilities in predicting 1-, 3-, and 5-year survival probabilities. Finally, *ERG* and *MTURN* were downregulated in LUAD samples, whereas *FOXM1* and *PTPR* were upregulated. Their expression levels were related to LUAD prognosis.

**Conclusion:**

*ERG*-cg27071152-*MTURN* and *FOXM1*-cg19212949-*PTPR* regulatory axes were proposed as potential biomarkers for predicting the prognosis of LUAD.

**Highlights**

• By integrating transcriptome and methylome profiles, the TF-CpG-gene triplet network was constructed.
• The prognostic models based on TF-CpG-gene axes show potential in predicting LUAD prognosis.
• Expression of TFs (*ERG* and *FOXM1*) and target genes (*MTURN* and *PTPR*) is related to LUAD prognosis.

## 1. Introduction

Lung adenocarcinoma (LUAD) accounts for the largest number of confirmed cases of non-small cell lung cancer, a pathological subtype of primary lung cancer, and is regarded as an aggressive tumor with an unfavorable prognosis.^[1,2]^ Relevant epidemiological data suggest that LUAD has an average 5-year survival rate of <20% even after conventional therapy, molecule-targeted therapy, and immunotherapy.^[3,4]^ Furthermore, the development of tumors is often accompanied by a series of complex genetic biological processes; gene function is the basis for supporting the clinical characteristics, and tumor-specific genes can be associated with clinicopathological parameters and predict tumor progression.^[5]^ From the perspective of genetics, the pathogenesis of LUAD depends on the activity of specific oncogenes.^[6]^ Therefore, many studies have been devoted to mining the prognostic signals of LUAD based on genomic and transcriptome data.^[7,8]^ However, only a fraction of patients have benefited, and more somatic genetic mechanisms, such as epigenetic modifications, should be considered in conjunction with transcriptomics to develop valuable prognostic biomarkers.

DNA methylation is a common epigenetic modification in mammalian genomes and often occurs on CpG islands. Abnormal methylation leads to changes in cellular function and can regulate carcinogenesis and metastasis of LUAD.^[9,10]^ As a relatively stable biochemical modification, DNA methylation can be detected in free DNA in tissues, serum, and plasma, and marker recognition based on methylation level therefore has potential clinical application prospect.^[11]^ Considerable evidence indicates that methylation-related biomarkers can be used for the early diagnosis and prognostic prediction of LUAD. Studies have assessed the methylation levels of 4 CpG loci of HOXA9, KrTAP8-1, CCND1, and TULP2 in LUAD samples and found that their methylation levels have great potential for the identification of uncertain LUAD nodules.^[12]^ Xu et al reported 33 methylation sites as specific prognostic biomarkers of LUAD based on resource processing from a public database.^[13]^ From the bioinformatics analysis of 6 methylation sites, a relevant study identified the prognostic features of LUAD, the signature of which is involved in protein binding and cytoplasm.^[14]^ Furthermore, it has been proposed that site-specific regulation of DNA methylation is mediated by DNA-bound transcription factors (TFs), which interact with specific promoter or enhancer regions.^[15]^ Abnormal methylation may affect tumor progression and prognosis through downstream transcriptional regulation.^[16]^ Studies have shown that gene promoters bound by Sp1, NRF1, and YY1 transcription factors can protect CpG island gene promoters from tumor-specific methylation.^[17]^ Differentially expressed methylation driver genes in LUAD were also found to be activated in processes such as DNA-binding transcriptional activator activity.^[18]^ However, the relationship between TF, methylation level, and gene expression in LUAD has not been clearly explained, and whether the regulatory relationship among them influences the prognosis of LUAD patients is also limited.

**Figure 1.**
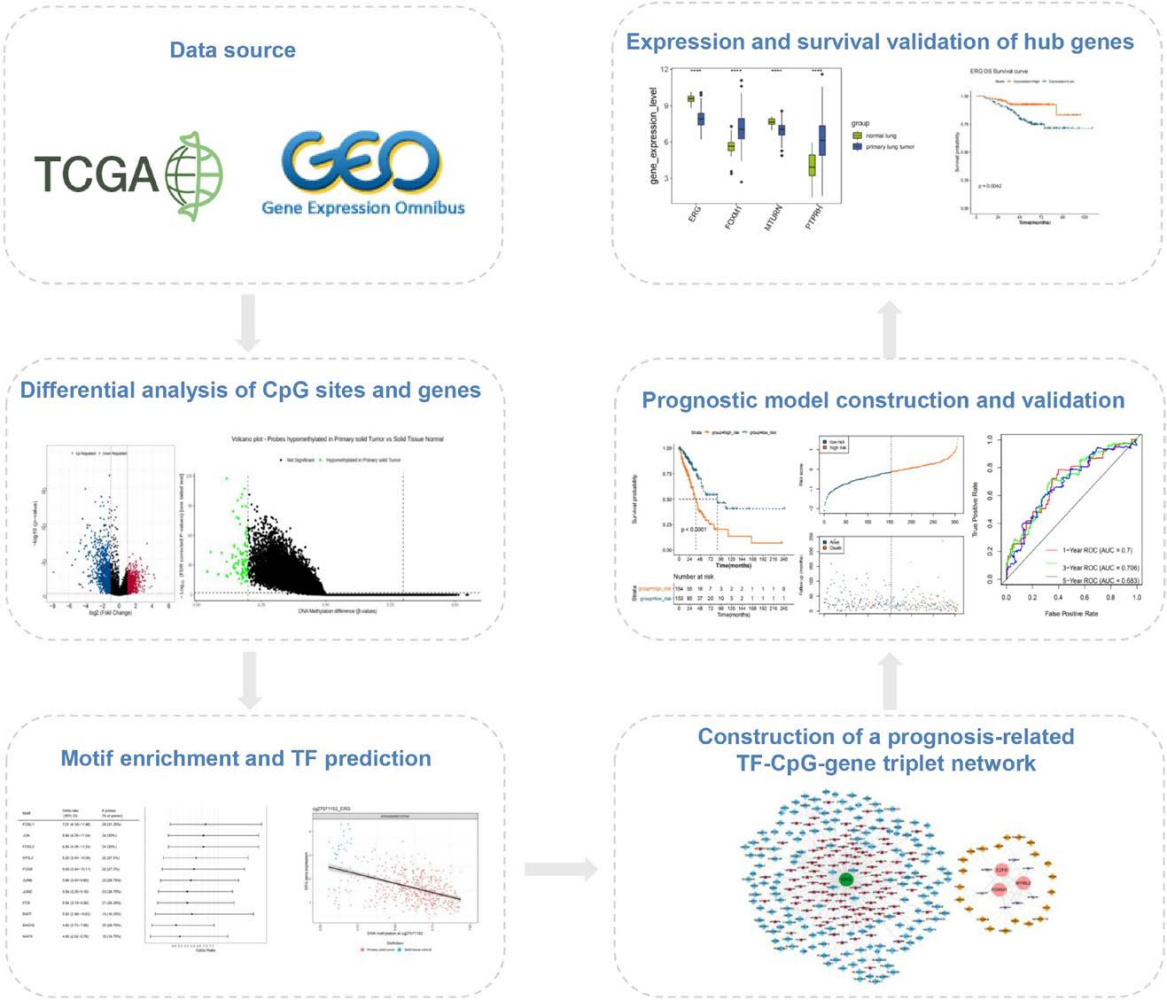
The workflow of this study.

Therefore, this study aimed to explore the specificity of TFs, methylation modification, and gene expression in LUAD, and explore the effect of TF-methylation-gene regulation on the prognosis of LUAD. As described in the study workflow (Fig. 1), the methylation data and transcriptome data from the sources of public databases were integrated in this study. After differential analysis, motif enrichment, TF prediction, and univariate Cox regression analysis, a prognostic TF-methylation-gene triplet network was constructed, followed by the establishment of a prognostic model. Along with internal and external validation, the predictive performance of the prognostic model based on the TF-methylation-gene regulatory axes was verified. Our study proposed potential prognostic markers for LUAD and provided new perspectives for understanding their regulatory mechanisms at the epigenetic level.

## 2. Methods

### 2.1. Data acquisition and processing

The methylation level and expression profile of TCGA-LUAD samples were downloaded from the genomic data commons hub of the UCSC-Xena platform (https://toil.xenahubs.net).^[19]^ Methylation data with pre-processed β signals were obtained using Illumina Infinium Human Methylation 450 Bead Chip, while the normalized log (fragments per lilobase per million + 1) expression data were detected using Illumina HiSeq 2000 RNA Sequencing. After matching sample numbers, a total of 450 LUAD and 21 normal samples with methylation levels and expression profiles were obtained, of which 439 tumor samples had prognostic information of overall survival (OS) and OS time. Meanwhile, the microarray data of GSE31210 were downloaded from the Gene Expression Omnibus database.^[20]^ This dataset contains expression data and survival information of 226 tumors and 20 normal samples for further external validation.

### 2.2. Differential analysis of gene expression and methylation level

The R3.6.1 limma V3.34.0^[21]^ was used to screen differential expressed genes (DEGs) between the tumor and control subjects with thresholds of |log (fold change)|>1 and adjusted *P* value (Benjaminiand & Hochberg method) < 0.05. For the *β* matrix of methylation sites, 2000bp methylation sites upstream and downstream of the transcription start site were extracted. The supervised method in the R.ELMER package (version 2.10.0, https://bioconductor.org/packages/release/bioc/html/ELMER.html)^[22]^ was used to select differential methylation sites (DMSs). The statistical significance of hyper-DMS/hypo-DMS was set at adjusted *P* < .05 and ⊿*β* > 0.3/⊿*β* < -0.3, respectively.

### 2.3. Identification of target genes of DMSs

Based on the DMSs obtained above, the ELMER package was used to screen potential target genes. After scanning the hyper-DMSs and hypo-DMSs, the top 10 DEGs with the nearest upstream and downstream loci were selected for the correlation analysis of methylation probes and target genes. With the thresholds of adjusted *P* < .05 and false discovery rate < 0.01, probe-gene relationship pairs were obtained.

### 2.4. Motif enrichment analysis and TF identification

Methylation probes in the probe-gene relations were used for enrichment analysis, and base sequences of the 250bp region upstream and downstream of the probes were extracted to map the enriched motif. Statistical significance was set at min.incidence = 10, lower odds ratio = 1.1, and false discovery rate < 0.05. Motif-binding TFs were predicted. Differentially expressed TFs that were negatively correlated with the methylation sites were selected. Because multiple TFs can be mapped on 1 motif, the top 5% differential TFs were selected for subsequent analysis.

### 2.5. Construction of the prognostic TF-methylation-gene network

The TF-methylation-gene relations were then obtained by integrating the TF-methylation and probe-gene relations. Combined with the prognostic information, the univariate Cox regression analysis in the R3.6.1 survival package (version 2.41-1, http://bioconductor.org/packages/survivalr/)^[23]^ was generated, and the TFs, methylation sites, and target genes (*P* < .05) were considered to have significant prognostic correlations. Then, the prognostic TF-methylation-gene relation pairs were obtained, and their regulatory networks were visualized using Cytoscape3.6.1.^[24]^ For the upregulated and downregulated genes in this network, DAVID version 6.8^[25,26]^ was used for gene ontology function and Kyoto encyclopedia of genes and genomes (KEGG) pathway enrichment analysis with a cutoff of *P* value < 0.05.

### 2.6. The prognostic model construction and validation

For each prognostic TF-methylation-gene relation pair, a multivariate Cox regression analysis was performed. The risk score-based prognostic model for each relation pair was constructed using the following formulas: risk score = ∑α*TF* + *βMeth* + *γGene*, where *α*, *β*, and *γ* represent the regression coefficients of TFs, methylation sites, and target genes, respectively. Receiver operator characteristic (ROC) curves were created and the area under the curves (AUCs) were calculated to predict the 1-, 3-, and 5-year survival rates. The 2 TF-methylation-gene triplets with the largest AUCs in hypomethylation and hypermethylation states were identified as the optimal prognostic models. To verify the model performance, the risk scores of each sample in the training and testing sets (with a ratio of 7:3) were calculated, and the samples were grouped into high-risk and low-risk groups using the medium values of the risk scores. The Kaplan-Meier (KM) method in the R3.6.1 survival package was used to assess the association between risk groups and actual survival status.

### 2.7. Expression and survival validation of hub genes

To verify whether the TFs and genes in the 2 optimal prognostic models were correlated with the prognosis and differentially expressed between tumor and normal samples, the GSE31210 dataset was employed. To evaluate the difference in expression levels of TFs and target genes, a *t* test was used, and statistical significance was set at *P* value < 0.05, followed by the creation of the box plot. The samples were also divided into high- and low-risk groups, and KM curves were generated using the log-rank test to assess the significance of differences in survival probability.

The overall workflow is shown in Figure 1.

## 3. Results

### 3.1. Differential analysis on methylation sites and gene expression

**Figure 2.**
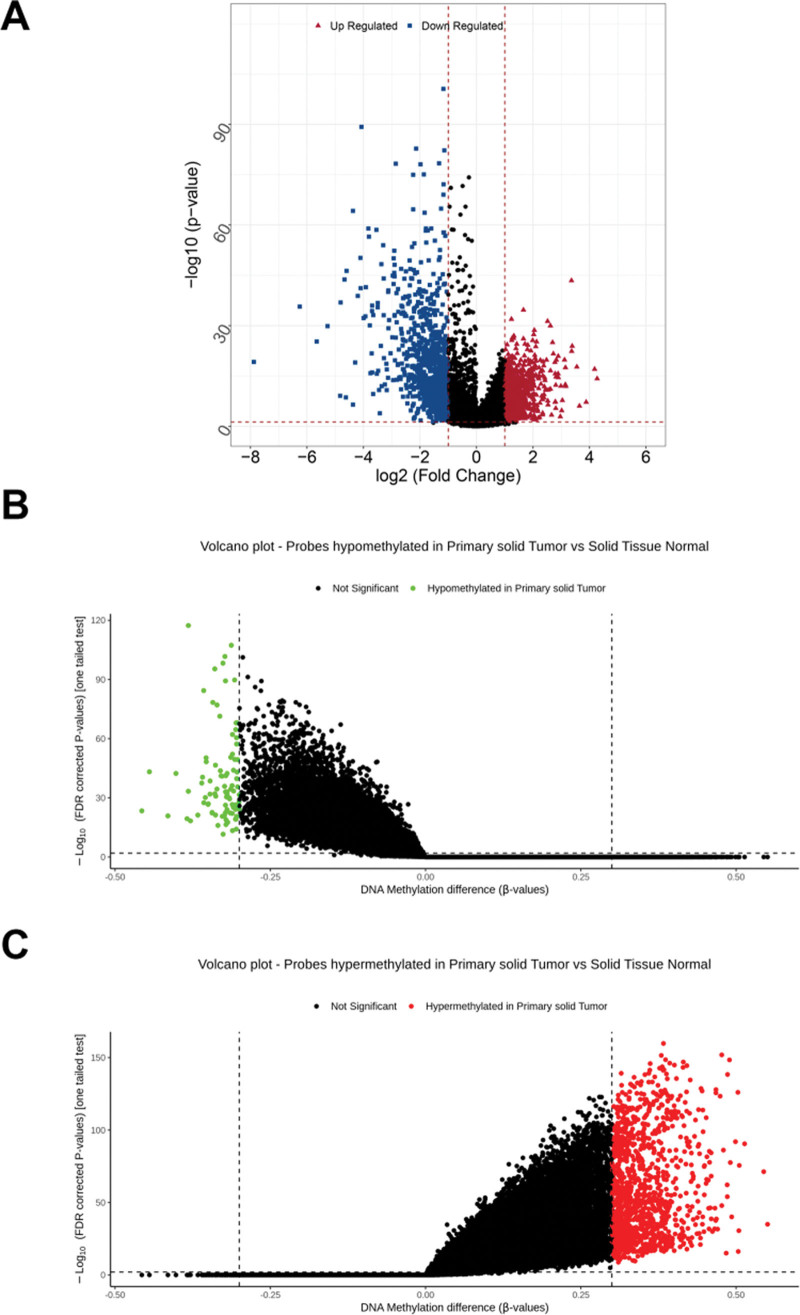
Differential analyses of genes and methylation sites. (A) Volcano plot showing the 873 upregulated DEGs and 1005 downregulated DEGs between LUAD and normal samples. (B and C) Volcano plots indicating the 90 hypo-DMSs (B) and 1143 hyper-DMSs (C) between LUAD and normal samples. DEG = differential expressed genes, DMS = differential methylation sites, LUAD = lung adenocarcinoma.

Based on the set thresholds, differential gene analysis was performed on 450 LUAD and 21 normal samples, and a total of 873 upregulated DEGs and 1005 downregulated DEGs were screened, as shown in Figure 2A. After the differential analysis of methylation sites using the ELMER package, a total of 90 hypo-DMSs (Fig. 2B) and 1143 hyper-DMSs (Fig. 2C) were obtained. The results indicate that the large number of hyperpromoter sites in LUAD may lead to downregulation of the genes and, in turn, promote disease progression.

### 3.2. Identification of target genes of DMSs

**Figure 3.**
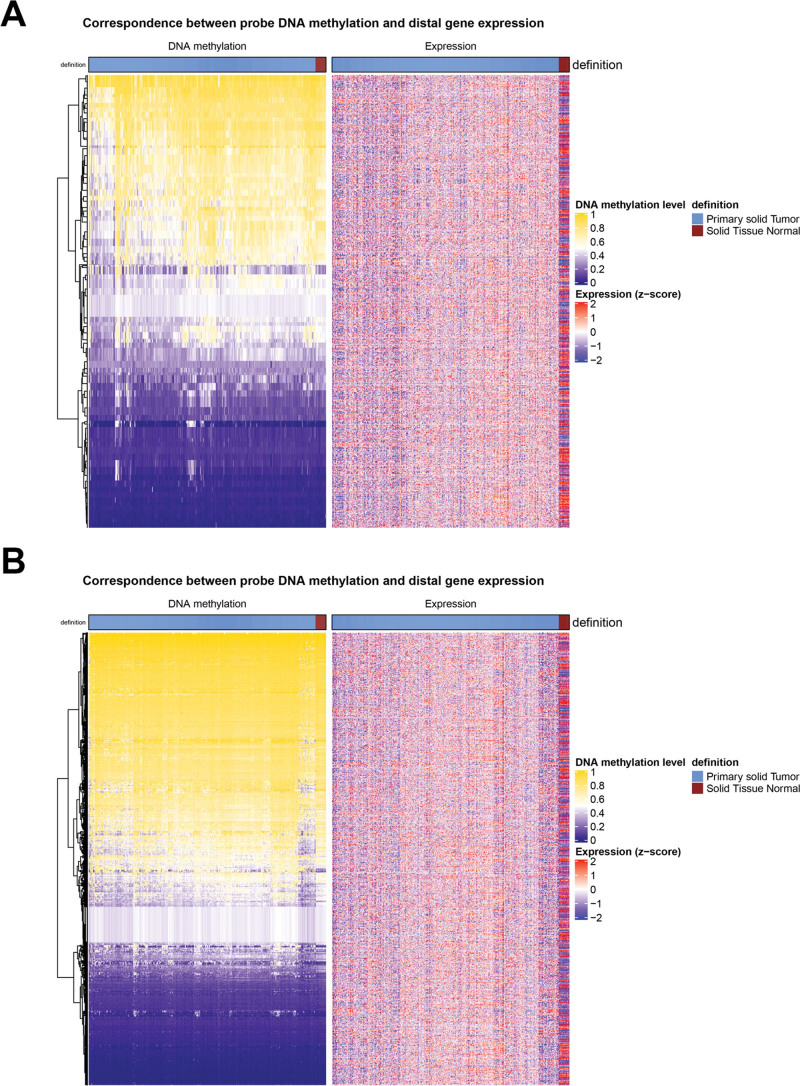
Identification of target genes of differential methylation sites. A-B: The heatmaps show the 784 hypo-gene relations (A) and 11206 hyper-gene relations (B) by considering the negative correlation between methylation sites and target genes.

The 20 upstream and downstream 20 DEGs closest to each hyper- and hypo-DMS were selected as target genes, and the correlations between methylation sites and target genes were analyzed. After selecting the negative correlation pairs, a total of 784 hypo-gene relations (including 80 hypomethylation probes and 356 target genes) and 11206 hyper-gene relations (including 1066 hypermethylation probes and 945 target genes) were selected (shown in Fig. 3A and B, respectively).

### 3.3. Motif enrichment analysis and TF prediction

**Figure 4.**
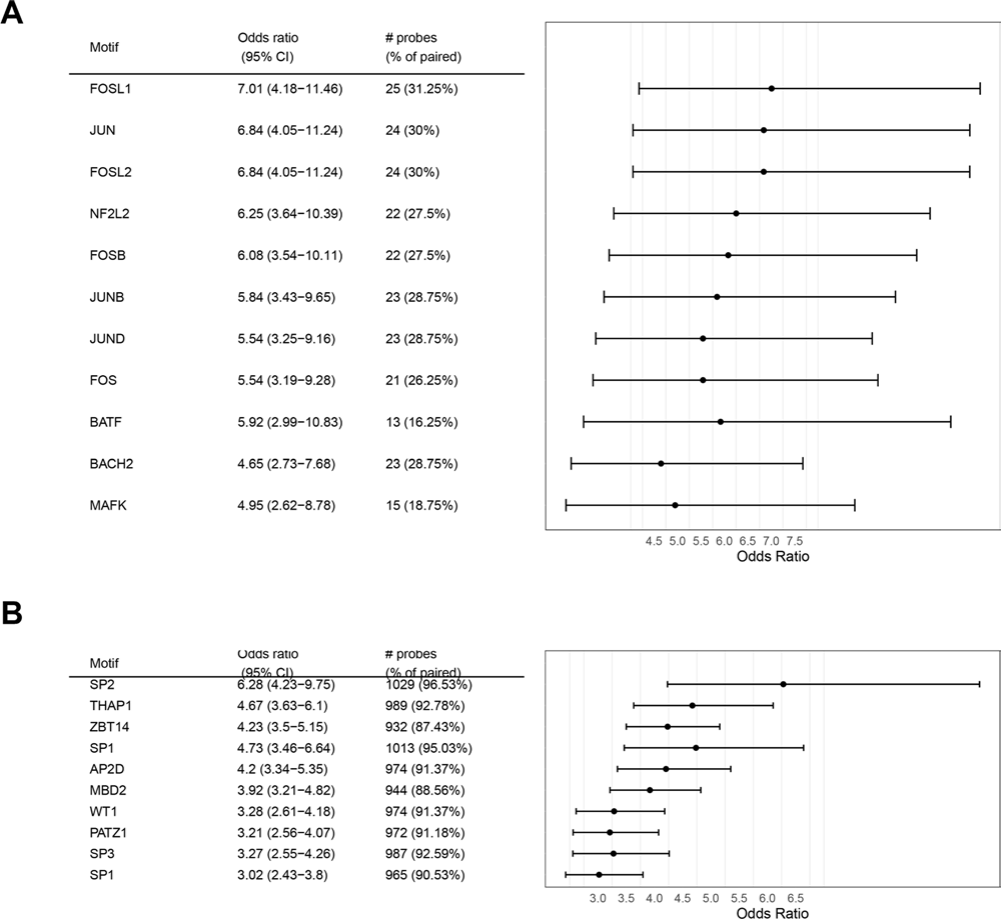
Motif enrichment analysis of methylation probes in methylation-gene relations. The top 10 enriched motifs of hypo- (A) and hypermethylation (B) based on the ranking of low OR values. OR = odds ratio.

The methylation probes in the methylation-gene relation pairs obtained above were used for further enrichment analysis. A total of 36 and 108 motifs were enriched in 778 hypo-gene relations and 11143 hyper-gene relations, respectively. According to the ranking of low odds ratio, the top 10 enriched motifs of hypo- and hypermethylation are displayed in Figure 4A and B. Furthermore, TFs that bind to motifs and are negatively correlated with methylation probes at the transcriptional level were predicted. The results suggested that *E2F8*, *ETV4*, *FOXM1*, *OTX1*, and *MYBL2* were predicted based on the motif enriched by hypomethylation probes, while *EPAS1*, *TAL1*, *ERG*, *SOX17*, and *SOX7* were candidate TFs for hypermethylation probes.

### 3.4. Construction of the prognosis-related TF-methylation-gene triplet network

**Figure 5.**
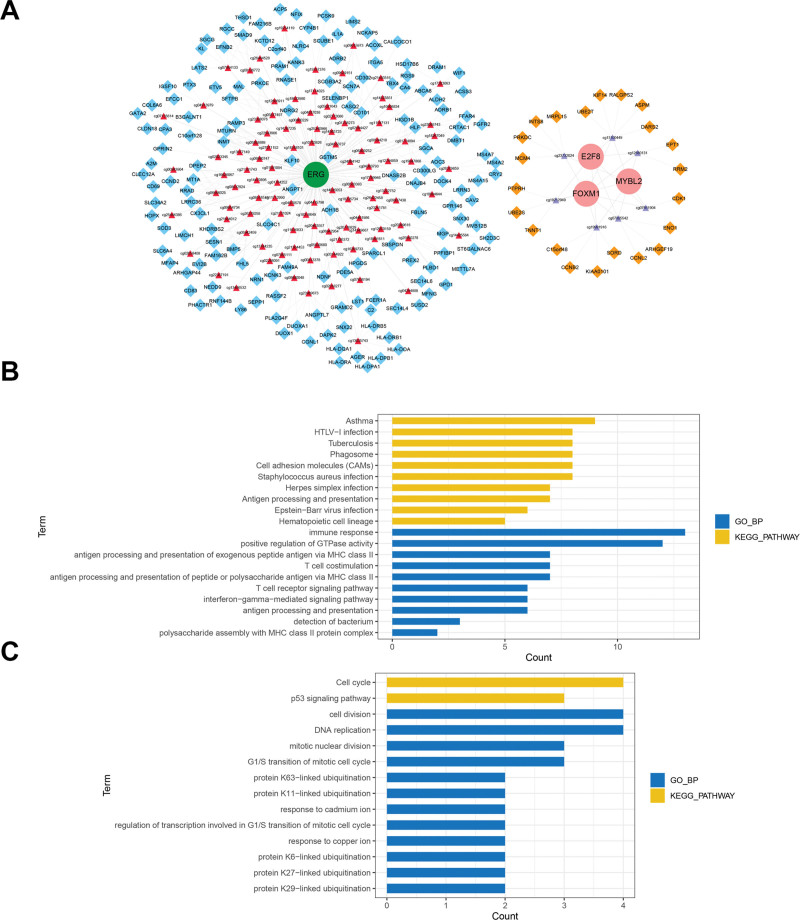
Construction of TF-methylation-gene triplet network and enrichment analysis of target genes. (A) The network contains 340 TF-hypergenes, and 66 TF-hypo-gene relation pairs were constructed. The circles represent TFs, and pink and green indicate upregulation and downregulation, respectively. The triangles represent the methylation probes, and the red and light purple indicate hypermethylation and hypomethylation sites, respectively. The diamonds represent target genes, and yellow and blue indicate upregulated and downregulated genes, respectively. (B and C) Gene ontology function and pathway enrichment analysis of down-regulated genes in the TF-hyper-gene network (B) and upregulated genes in the TF-hypo-gene network (C). TF = transcription factors.

The TF-methylation-gene triplets were obtained by integrating the TF-methylation and methylation-gene relationships. After generating the univariate Cox regression analysis of TF, methylation probes, and target genes, a total of 4 TFs, 111 methylation sites, and 177 target genes were found to be significantly correlated with prognosis. Then, a network comprising 340 TF-hyper-gene and 66 TF-hypo-gene relation pairs was constructed, as shown in Figure 5A. In this network, *ERG* is the hub TF in the TF-hyper-gene relations, while *E2F8*, *FOXM1*, and *MYBL2* are involved in TF-hypo-gene relations. Furthermore, enrichment analysis was performed on the upregulated genes in the TF-hypo-gene and down-regulated genes in the TF-hyper-gene network. As a result, the downregulated genes were mainly enriched in the biological process of cell cycle, as well as in the KEGG pathways of cell decision and DNA replication (Fig. 5B). The upregulated genes in the TF-hyper-gene network were mainly enriched in the gene ontology-biological process of asthma and the KEGG pathways of immune response and positive regulation of GTPase activity (Fig. 5C).

### 3.5. Construction and validation of the prognostic model

**Figure 6.**
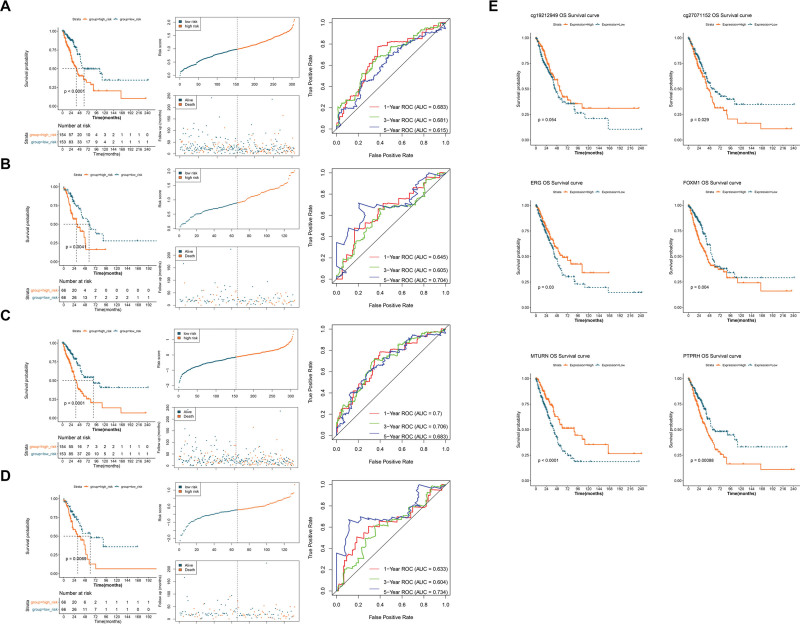
Establishment and validation of the prognostic based on the optimal TF-methylation-gene pairs. (A and B) Construction and validation of the prognostic model based on the *FOXM1*-cg19212949-*PTPR*H pair in the training set (A) and testing set (B). (C and D) Construction and validation of the *ERG*-cg27071152-*MTURN*-related prognostic model in the training set (C) and testing set (D). E: The KM curves show the relationship between survival prognosis and the methylation level and expression levels of the components of the prognostic models. KM = Kaplan-Meier, TF = transcription factors.

As described in the Section 2, multivariate Cox regression analysis was performed on each TF-methylation-gene pair. The optimal down-hyper-down (*ERG*-cg27071152-*MTURN*) and up-hypo-up (*FOXM1*-cg19212949-*PTPR*H) pairs were selected based on their AUC ranking, to further be involved in the risk score-based prognostic models. Then, the samples of TCGA were divided into the training set and testing set at a ratio of 7:3, and the multivariate Cox regression coefficients of the above 2 TF-methylation-gene pairs in the training set were calculated, as listed in Table 1. The samples were further divided into risk groups according to their respective median risk scores. The results of the prognostic model constructed using the *FOXM1*-cg19212949-*PTPR*H pair suggested that patients in the high-risk group had worse survival status in both the training set (Fig. 6A) and testing set (Fig. 6B). It was also found that high-risk patients in the *ERG*-cg27071152-*MTURN*-related prognostic model had worse OS rates than those in the low-risk group in the training set (Fig. 6C) and testing set (Fig. 6D). Furthermore, the AUCs of these 2 models in the training set and testing set were all >0.6 (Figs. 6A–D), thereby indicating that they have good predictive performance for LUAD prognosis. Based on the expression levels of TFs and genes, as well as the methylation levels in the 2 prognostic models, KM curves were created to evaluate the relationship between these components and survival prognosis. The results (Fig. 6E) suggested that patients with high expression of *FOXM1* and *PTPR*H, as well as high methylation levels of cg27071152, had worse prognosis. However, patients with high expression of *ERG* and *MTURN*, as well as high methylation levels of cg19212949, had superior clinical outcomes.

**Table 1:** The multivariate Cox regression coefficients of TFs, methylation probes, and genes in the two prognostic models.

Components	Coef	Up_down
*FOXM1*	0.253	Up
cg19212949	–0.219	Hypo
*PTPRH*	0.215	Up
*ERG*	0.004	Down
cg27071152	1.993	Hyper
*MTURN*	–0.550	Down

Hypo = hypomethylation, Hyper = hypermethylation, TF = transcription factors.

### 3.6. Expression and survival validation of hub genes

**Figure 7.**
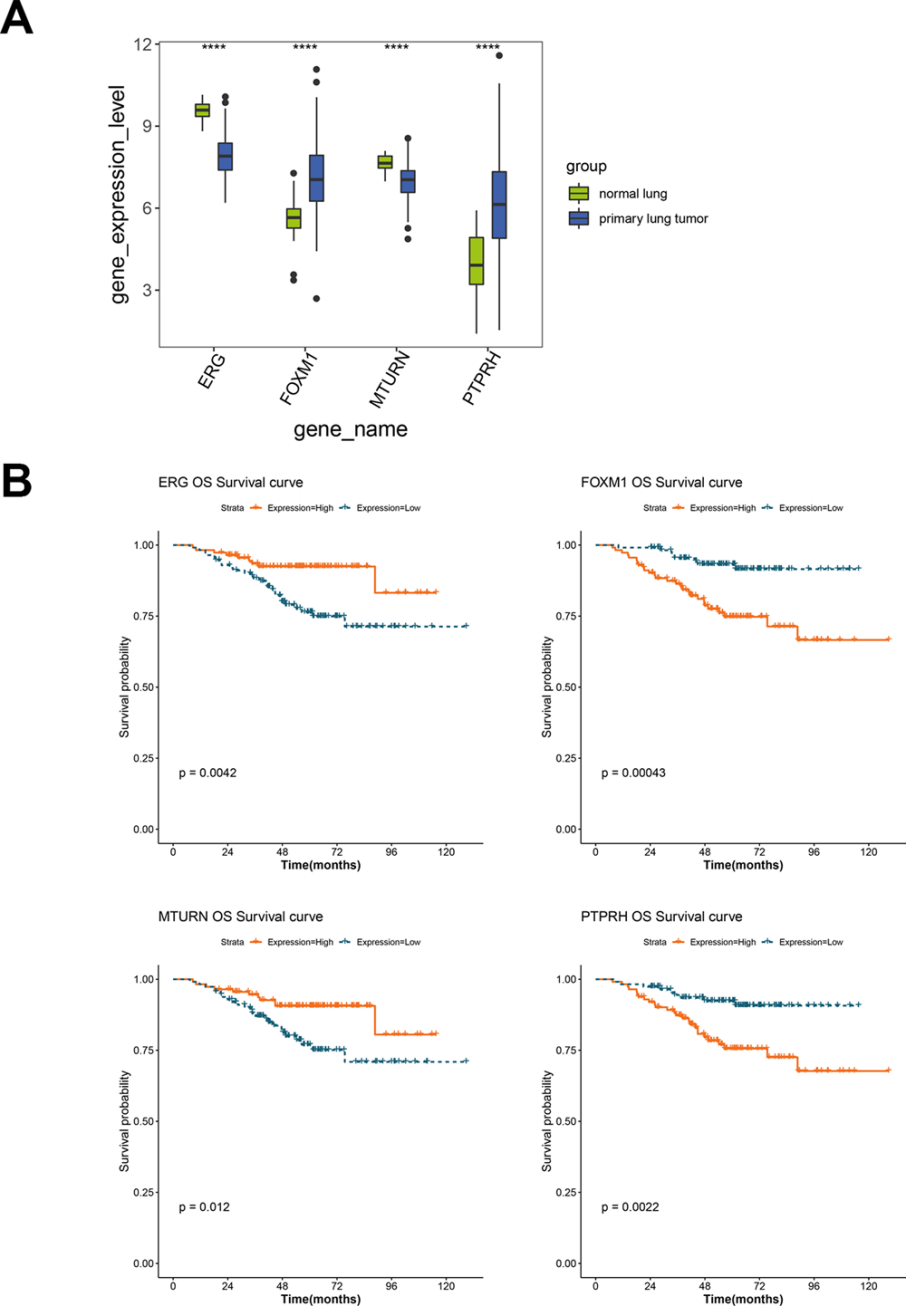
External validation of expression and survival of hub genes and TFs. (A) The expression difference of the four TFs and genes between primary lung tumors and normal tissues from the GSE31210 dataset. (B) The KM curves show the association between the survival prognosis of LUAD and the expression of hub TFs and genes. LUAD = lung adenocarcinoma, TF = transcription factors.

The expression data of TFs (*FOXM1* and *ERG*) and genes (*PTPR*H and *MTURN*) were extracted from the GSE31210 dataset, and box plots were created to validate their expression. As shown in Figure 7A, the 4 TFs and genes were all significantly differentially expressed between primary lung tumors and normal tissues (*P* < .001), and their expression trends were consistent with the TCGA results. The results of the survival validation (Fig. 7B) suggested that high expression of *FOXM1* and *PTPR*H, as well as the low expression of *ERG* and *MTURN*, were related to an unfavorable prognosis, which was also in accordance with the results based on TCGA samples.

## 4. Discussion

Bioinformatics is an approach to systematically and comprehensively decipher complex tumor pathogenesis through the integration of multiple omics techniques. Therefore, bioinformatics analysis was used in this study to mine 1878 DEGs and 1233 DMSs between LUAD and normal tissue samples. The differential analysis of genes and methylation sites is usually the first stage to identify the prognostic features, and these DEGs and DMSs are believed to be LUAD-specific and play important roles in tumor progression, diagnosis, and prognosis.^[27]^ Considering that the mining of upstream regulatory factors of these genes will facilitate the exploration of LUAD-related epigenetic regulatory mechanisms, we identified the target genes of DMSs. This was followed by motif enrichment in the promoter region and the prediction of TFs. It was found that abundant motifs were enriched in the upstream and downstream regions of the methylation site, and that they can also be bound by TFs. Epigenetic modifications regulate the dynamic combination of TFs and regulatory elements, thus controlling the transcription state during tumor progression.^[28]^ Based on the TF-methylation and methylation-gene relation pairs, we constructed the TF-methylation-gene axes to further explore their prognostic value.

By generating the univariate Cox regression analysis, a prognostic TF-methylation-gene triplet network was constructed, which contained 4 TFs, 111 methylation sites, and 177 target genes. Among them, the downregulated genes were mainly enriched in the immune response pathway. Studies have indicated that the degree of tumor malignancy is associated with the tumor microenvironment (TME), which mainly consists of immune cells, cytokines, chemokines and extracellular matrix molecules, and the LUAD is in the dynamic communication with the surrounding TME.^[29,30]^ Tumor cells can evade immune surveillance of the host and provide the possibility for their proliferation.^[4]^ Numerous studies have identified the immune characteristics of LUAD to provide clinical guidance for the diagnosis and prognosis of LUAD with different immunophenotypes.^[30,31]^ Our study also identified some downregulated genes in LUAD as tumor suppressors, whose abnormal expression may cause ineffective immune response and provide opportunities for immune escape of tumor cells, thus affecting the prognosis of LUAD. Furthermore, the upregulated genes in this TF-methylation-gene network were mainly enriched in cell cycle-related biological processes and pathways, such as cell division and DNA replication. Cell cycle controller dysfunction disrupts the highly regulated mitosis process, while blocking mitosis and chromosomal instability will lead to faulty DNA replication, thereby further leading to somatic mutations and tumorigenesis.^[32,33]^ Relevant studies have predicted and confirmed the independent prognostic roles of several cell cycle-related genes in non-small cell lung cancer.^[34,35]^ The genes involved in cell cycle-related pathways identified in this study are oncogenes that are upregulated in LUAD, which may hinder DNA replication and cell division and affect the clinical outcome of LUAD patients by promoting tumor recurrence.

Based on the TF-methylation-gene triplet network, we selected *ERG*-cg27071152-*MTURN* and *FOXM1*-cg19212949-*PTPR* regulatory axes to further establish the prognostic models that both showed robust abilities in predicting the prognosis of LUAD. Among them, *ERG* and *FOXM1* were considered core TFs. *ERG* can promote endothelial homeostasis by regulating family specific enhancers, and the abnormal expression of *ERG* in cancer is carcinogenic.^[36]^ Our results also confirmed that the downregulation of *ERG* was significantly associated with the occurrence and prognosis of LUAD. We also interpreted *FOXM1* as a prognostic risk factor, with higher expression associated with lower survival probability. *FOXM1* has been proven to be a proto-oncogene that can drive the activation of lung fibroblasts.^[37]^ The high expression of *FOXM1* in cancer can activate the nuclear transcription of cell cycle regulation genes,^[38]^ which may affect the role of its target gene *PTPR* in cell division and DNA replication. *PTPR* is also considered to be carcinogenic, which is consistent with our results, and its family gene *PTPRZ1* can regulate calmodulin phosphorylation and tumor progression in small cell lung cancer.^[39]^ Moreover, the results of internal and external validation in this study suggested that *MTURN* was downregulated in LUAD samples. This finding was supported by Liu et al, and *MTURN* may serve as a potential biomarker for blood-based lung cancer diagnosis, as well as the prediction of chemotherapy response.^[40]^ The transcriptional regulatory mechanisms between *ERG* and *MTURN*, as well as *FOXM1* and *PTPR*, are limited, but our results suggest that *ERG*-*MTURN* and *FOXM1*-*PTPR* may be regulated by methylation at cg27071152 and cg19212949, respectively.

Due to the lack of clinical information on LUAD samples, the inability to stratify the samples based on tumor grade and TNM stage was 1 of the limitations of this study. Therefore, the roles of *ERG*-cg27071152-*MTURN* and *FOXM1*-cg19212949-*PTPR* in different tumor stages and in LUAD progression were explored. In the follow-up study, solid tumor samples were collected to verify the transcriptional regulatory relationship of candidate genes through experiments. Meanwhile, more detailed information of patients with LUAD will be captured to associate the candidate regulatory axes with clinicopathological parameters and explore the impact of TFs and target genes on clinical manifestations.

## 5. Conclusion

By integrating transcriptomic and methylation data, a TF-methylation-gene triplet network significantly associated with LUAD prognosis was constructed in this study. The prognostic models constructed based on *ERG*-cg27071152-*MTURN* and *FOXM1*-cg19212949-*PTPR* axes from the network showed the potential to predict the prognosis of LUAD. This study proposed several valuable prognostic biomarkers for LUAD and provided a theoretical basis for customizing personalized therapeutic strategies to improve the clinical outcomes of patients with LUAD.

## Author contributions

**Conceptualization:** Duohuang Lian, Luoyu Lian, Wenmin Ying, Shunkai Zhou.

**Data curation:** Duohuang Lian, Luoyu Lian.

**Formal analysis:** Dehua Zeng.

**Methodology:** Dehua Zeng.

**Project administration:** Meiqing Zhang.

**Resources:** Meiqing Zhang.

**Software:** Mengmeng Chen.

**Validation:** Mengmeng Chen, Yaming Liu.

**Visualization:** Yaming Liu.

**Writing – original draft:** Duohuang Lian, Luoyu Lian.

**Writing – review & editing:** Wenmin Ying, Shunkai Zhou.
